# Identification and clinical validation of gene signatures with grade and survival in head and neck carcinomas

**DOI:** 10.1590/1414-431X2020e11069

**Published:** 2021-09-20

**Authors:** Wei Ma, Qing Cao, Wandong She

**Affiliations:** 1 Department of Otolaryngology - Head and Neck Surgery, Nanjing Drum Tower Hospital Clinical College of Nanjing Medical University, Nanjing, Jiangsu, China; 2 Department of Otolaryngology - Head and Neck Surgery, Clinical Medical College, Yangzhou University, Yangzhou, Jiangsu, China

**Keywords:** Head and neck carcinoma, Malignancy, Grade, Prognosis, GEO

## Abstract

This study aimed to explore gene expression profiles that drive malignancy from low- to high-grade head and neck carcinomas (HNC), as well as to analyze their correlations with survival. Gene expressions and clinical data of HNC were downloaded from the Gene Expression Omnibus (GEO) repository. The significantly differential genes (SDGs) between low- and high-grade HNC were screened. Cox regressions were performed to identify prognostic SDGs of progression-free survival (PFS) and disease-specific survival (DSS). The genes were experimentally validated by RT-PCR in clinical tissue specimens. Thirty-five SDGs were identified in 47 low-grade and 30 high-grade HNC samples. Cox regression analyses showed that CXCL14, SLC44A1, and UBD were significantly associated with DSS, and PPP2R2C and SLC44A1 were associated with PFS. Patients were grouped into high-risk or low-risk groups for prognosis based on gene signatures. High-risk patients had significantly shorter DSS and PFS than low-risk patients (P=0.033 and P=0.010, respectively). Multivariate Cox regression showed HPV (P=0.033), lymph node status (P=0.032), and residual status (P<0.044) were independent risk factors for PFS. ROC curves showed the risk score had better efficacy to predict survival both for DSS and PFS (AUC=0.858 and AUC=0.901, respectively). The results showed CXCL14 and SLC44A1 were significantly overexpressed in the low-grade HNC tissues and the UBD were overexpressed in the high-grade HNC tissues. Our results suggested that SDGs had different expression profiles between the low-grade and high-grade HNC, and these genes may serve as prognostic biomarkers to predict survival.

## Introduction

Head and neck carcinomas (HNC) are a group of heterogeneous tumors arising from the oral cavity, oropharynx, nasopharynx, hypopharynx, and larynx, ranking as the sixth most prevalent cancer (1,2). Head and neck squamous cell carcinomas (HNSCC) account for 90% of all head and neck carcinomas. More than 600,000 new HNC cases and 350,000 deaths are estimated per year globally (3,4). HNC can be classified into subgroups according to the human papillomavirus (HPV) status and histological grades (2,4). In approximately 42% of patients, HNC is diagnosed in an advanced stage with extensive lymph nodes or distant metastasis at their initial visits (5). Patients with HNC have benefited from comprehensive treatment in recent years. However, low-grade HNC have different treatment modalities from the advanced ones, and the 5-year survival remains less than 50% despite the tremendous progress that has been made in the multidisciplinary treatment, including surgery, radiotherapy, and chemotherapy (6).

Selection of optimal management plans for HNC is mainly dependent on tailored risk evaluation (7,8). Histological grade in HNC helps to assess the patients' risks to make therapeutic strategies and provide important clinical prognostic information. Despite the significance, relying solely on histological grade cannot provide a reference for clinical decision-making owing to diagnostic inconsistence and classification discordance with different standards (9,10). Additionally, the underlying mechanisms regulating HNC progression from low- to high-grade, such as NF-κB pathways, are still largely unknown. Therefore, it is imperative to identify new methods and biomarkers for increasing pathological grade values along with discovering new mechanisms about the transition from low-grade towards high-grade. The extensive applications of high-throughput sequencing technologies in cancer biology, such as gene profile analysis, have revealed the relationship between thousands of aberrant gene expressions associated with HNC patients (11,12). Among those that have been functionally characterized, several have been linked to malignant progression (13,14). Notably, many genes have key roles for diagnostic accuracy and for predicting the prognosis (15,16).

In this study, we have comprehensively analyzed the significantly differential genes (SDGs) and clinical information from the Gene Expression Omnibus (GEO) in order to explore whether different grade HNC have distinct gene expressions. To determine the clinical relevance, we also investigated the associations between genes and survival. Results were further verified in experiments using clinical tissue specimens.

## Material and Methods

### Patient samples and data extraction

The gene expression data and clinical information of HNC were downloaded from GEO (https://www.ncbi.nlm.nih.gov/gds/). SDGs were obtained from GSE117973 and were initially analyzed with GEO2R. The R software (version 3.6.1) was used to identify SDGs using the Wilcoxon test with the “limma” package. In this dataset, the classification of low-grade and high-grade HNC was based on the TNM stage, in which patients with stage I/II were classified into low-grade HNC and stage III/IV into high-grade. The SDGs with false discovery rate (FDR) <0.05 and |log2 fold change (FC)| >0.5 were considered to be differentially expressed.

### Enrichment analysis

The functional analyses of Gene Ontology (GO) and Kyoto Encyclopedia of Genes and Genomes (KEGG) pathway were conducted using the SDGs with the R package. GO analysis includes the biological process (BP), cellular component (CC), and molecular function (MF). Top results with an FDR ≤0.05 were considered noteworthy.

### Survival analysis and ROC analysis

We evaluated the correlations between the disease-specific survival (DSS), progression-free survival (PFS), and SDGs by univariate and multivariate Cox proportional hazards regression analyses. The prognostic factors (P<0.05) were entered into multivariate Cox regression to identify the independent prognostic risk factors.

The receiver operating curve (ROC) analysis was used to assess the sensitivity and specificity of the independent risk factors. The area under the curve (AUC) of the ROC ranges from 0.5 to 1, with 1 indicating perfect predictive ability and 0.5 indicating no predictive ability.

### Experimental validation

To verify the prognostic genes expression levels in HNC tissues, we conducted the experimental validation in 45 specimens from patients with HNC (25 grade I/II and 20 grade III/IV) who underwent surgery from January 2019 to August 2020 at the Clinical Medical College of Yangzhou University, Yangzhou, Jiangsu. This study was approved by the Internal Review Board of the Clinical Medical College of Yangzhou University, Yangzhou, Jiangsu.

Total RNA from 45 HNC tissues was purified using RNAiso plus (Takara, China). Complementary DNA (cDNA) was synthesized from 1 μg of total RNA using a PrimeScript^®^ RT reagent kit with gDNA (genomic DNA) Eraser (Takara). TB Green^®^ Premix Ex Taq^®^ II kit (Takara) was used to detect the indicated RNA levels on the QuantStudio real-time polymerase chain reaction (PCR) system (Applied Biosystems, USA). One cycle of RT reaction was performed under the following conditions: 30°C for 10 min, 42°C for 30 min, 95°C for 5 min, and 5°C for 5 min. PCR was performed using a Takara Shuzo PCR amplification kit (cat. No. R011; Takara Bio, Inc., China) with primer sets specific for different genes. The thermal conditions for the gene and glyceraldehyde-3-phosphate dehydrogenase (GAPDH) were denaturation for 30 s at 95°C, annealing for 30 s at 56°C, and extension for 30 s at 72°C. The amplifications were performed using 25-28 cycles. The relative expression levels of the candidate genes were normalized to endogenous GAPDH. The primers were synthesized by GENEWIZ Co. (China). The primers are listed in Supplementary Table S1.

## Results

### Distinct gene patterns in low-grade and high-grade HNC tissues

**Figure 1:**
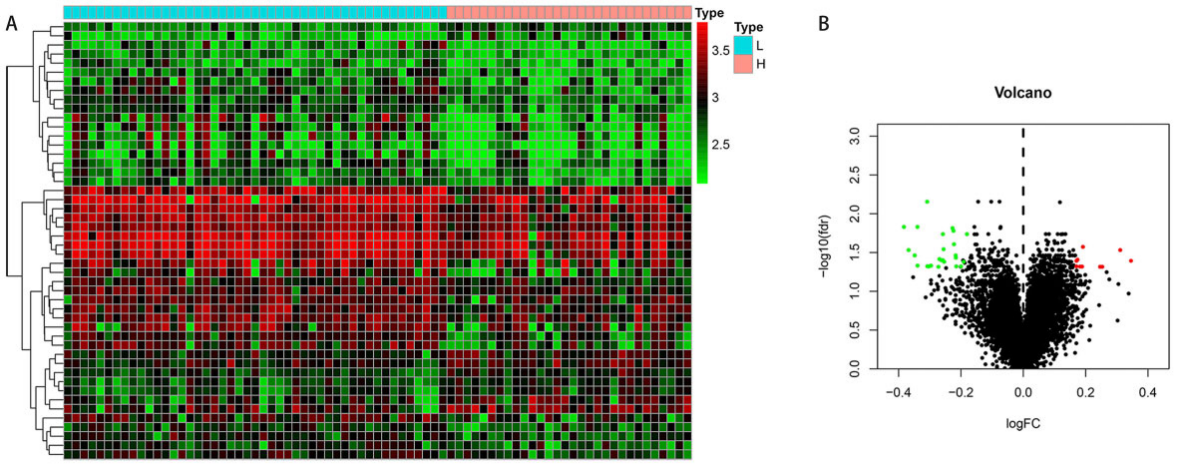
**A**, Heatmap of significantly differential genes (SDGs) expression profiles in low-grade and high-grade head and neck carcinoma (HNC) samples. The red color indicates the up-regulated genes and green indicates down-regulated genes. L: low-grade; H: high-grade. **B**, Volcano plot of SDGs in low-grade and high-grade HNC samples. The red dots represent upregulated genes, and the green dots represent downregulated genes.

A total of 77 HNC samples with gene expressions and clinical data were obtained from GSE117973, including 47 low-grade and 30 high-grade samples. There were 35 SDGs between the two groups. Among these SDGs, 23 genes were downregulated and 12 were upregulated in the high-grade group compared with low-grade group (Table 1). The heatmap and volcano plots are shown in Figure 1.

**Table 1:** Significantly differential genes (SDGs) expression levels in low- and high-grade head and neck cancer tissues.

Gene	Low-grade	High-grade	FDR	P value	logFC
ALPK1	6.431	6.977	0.020	6.00E-06	0.546
ATF5	8.712	9.569	0.020	4.70E-06	0.857
BICD2	7.048	6.691	0.026	1.58E-05	-0.691
CALB2	7.165	5.662	0.020	5.45E-06	-1.502
CXCL14	10.954	9.390	0.026	1.30E-05	-1.564
F2RL1	8.490	6.856	0.015	5.88E-07	-1.682
FAM117A	4.871	5.462	0.029	2.02E-05	0.591
FAM89A	7.345	6.590	0.020	3.83E-06	-1.045
GALNT1	8.069	7.514	0.024	1.09E-05	-0.555
GOLGA7B	5.701	5.030	0.024	1.10E-05	-1.101
HIF1A	7.608	7.047	0.036	3.19E-05	-0.67
KCNK1	8.244	7.343	0.029	1.97E-05	-0.886
KRT16	12.271	10.636	0.043	4.00E-05	-1.426
KRT16P2	11.462	9.547	0.048	5.22E-05	-2.09
KRT6B	10.555	9.037	0.020	2.69E-06	-1.729
LGALS9	5.684	6.491	0.026	1.38E-05	0.846
LIG1	7.338	8.007	0.029	2.33E-05	0.669
MALL	10.777	9.747	0.020	3.46E-06	-1.029
MAST3	6.610	7.192	0.026	1.64E-05	0.581
MID2	6.117	5.660	0.029	2.31E-05	-0.689
POLD1	7.083	7.734	0.026	1.45E-05	0.651
POLE	6.652	7.181	0.029	2.05E-05	0.529
PPP2R2C	6.728	5.749	0.024	7.74E-06	-1.989
PTHLH	8.803	6.751	0.020	2.97E-06	-2.052
RFX5	6.940	7.304	0.024	9.02E-06	0.663
RNF44	7.375	7.903	0.024	1.13E-05	0.528
S100A10	13.255	12.795	0.024	8.38E-06	-0.509
SC5D	8.958	8.035	0.020	1.93E-06	-0.923
SLC44A1	9.885	9.376	0.029	2.11E-05	-0.519
SNAPC1	5.596	5.046	0.048	5.14E-05	-0.55
TCF19	5.148	5.506	0.045	4.43E-05	0.553
TUBA4A	10.600	9.571	0.029	2.30E-05	-1.029
UBD	7.873	9.767	0.047	4.73E-05	1.894
UPP1	10.648	9.571	0.028	1.86E-05	-1.077
ZFAND2A	9.227	8.661	0.043	4.18E-05	-0.566

logFC: log fold change; FDR: false discovery rate.

### Enrichment analysis

**Figure 2:**
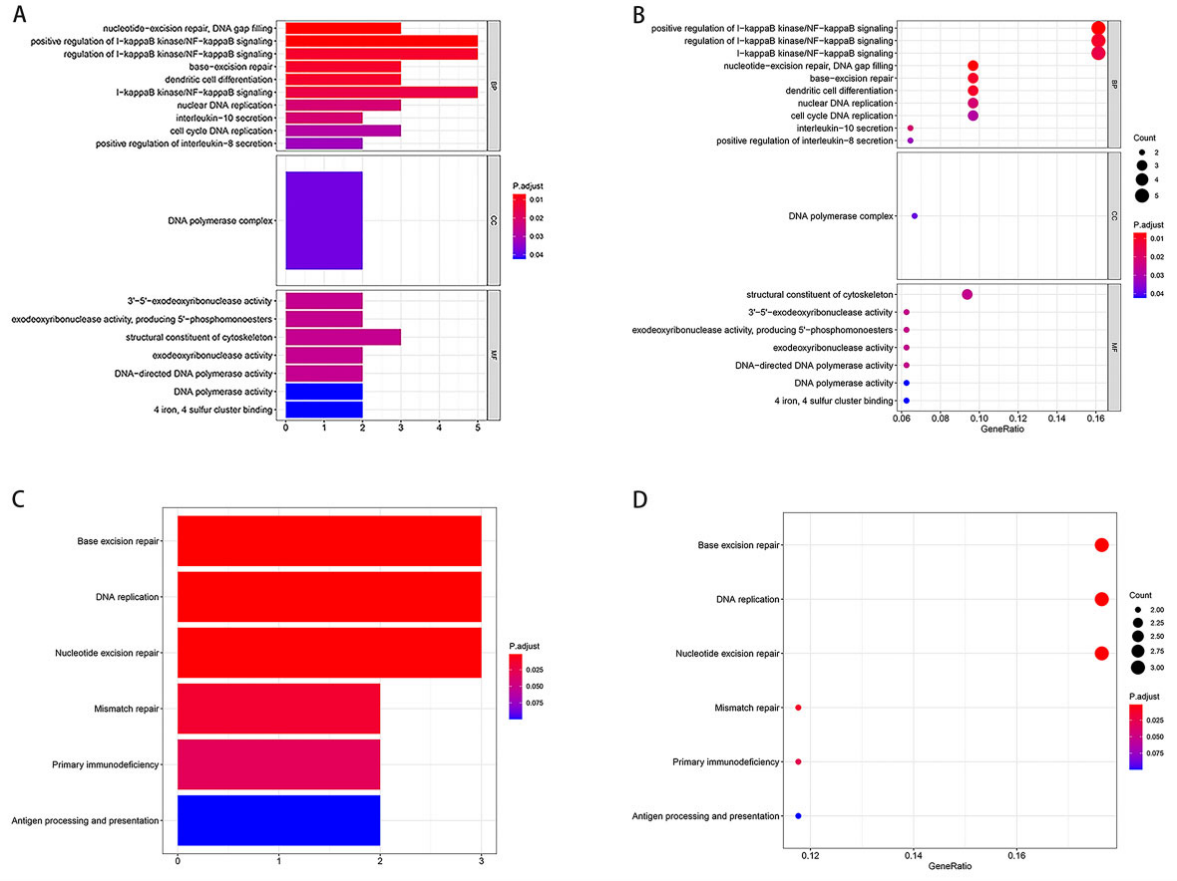
Enrichment analysis of significantly differential genes (SDGs). **A**, Bar plot of Gene Ontology (GO) analysis, including the biological process (BP), cellular component (CC), and molecular function (MF) analysis. **B**, Bubble diagram of GO analysis. Larger bubble and darker color indicate more a significant enrichment process. **C**, Bar plot of Kyoto Encyclopedia of Genes and Genomes (KEGG) analysis. **D**, Bubble diagram of KEGG analysis.

Given the importance of the SDGs and further exploration about their functions, we performed the GO and KEGG analysis. GO results showed that SDGs were strongly associated with nucleotide-excision repair and DNA polymerase complex pathways. In the BP category, the SDGs were enriched in the nucleotide-excision repair pathway, as well as the regulation of I−kappaB kinase/NF−kappaB signaling pathway. In the CC category, SDGs were enriched in the DNA polymerase complex pathway. In the MF category, the SDGs were enriched in the structural constituent of cytoskeleton (Figure 2A and B). In the KEGG analysis, SDGs were involved in the base excision repair activity, which was similar in the GO analysis (Figure 2C and D).

### Prognostic SDGs in DSS and PFS

**Figure 3:**
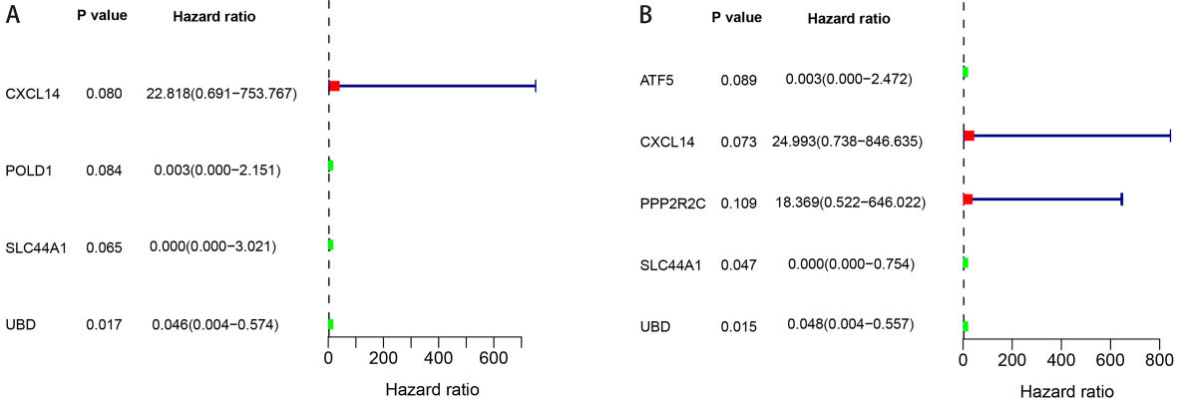
P values and hazard ratios of the risk of significantly differential genes (SDGs) for (**A**) disease-specific survival (DSS) and (**B**) progression-free survival (PFS) models are shown. Survival analysis shows UBD is significantly associated with DSS and SLC44A1 and UBD are significantly associated with PFS in the univariate Cox regression.

**Figure 4:**
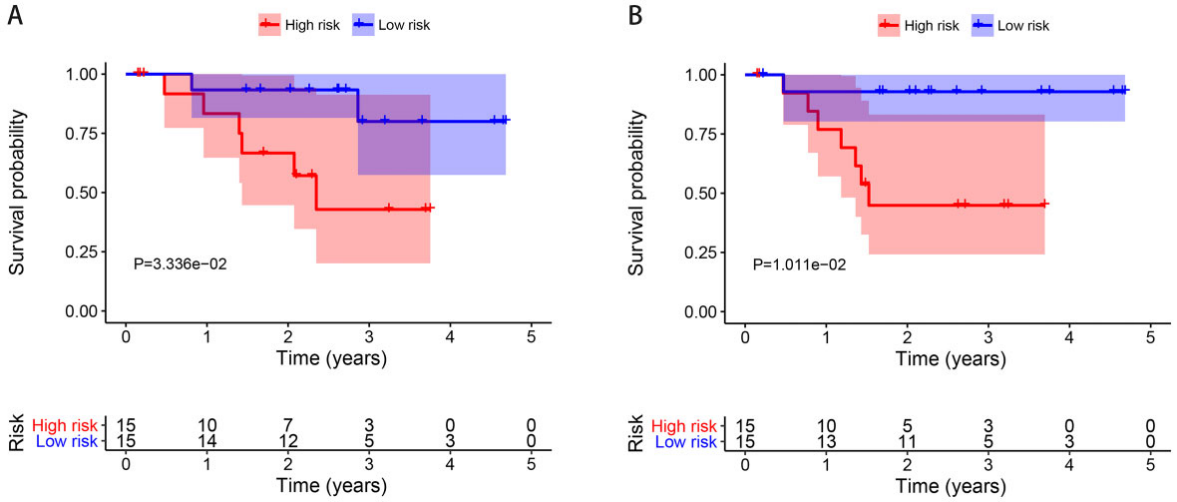
Kaplan-Meier curve for (**A**) disease-specific survival (DSS) and (**B**) progression-free survival (PFS) in the high-risk and low-risk head and neck carcinomas (HNC) patients when stratified by the risk score. Low-risk group patients had higher survival probabilities than those in the high-risk group (P=0.033 and P=0.010, respectively).

To explore whether the SDGs are associated with DSS and PFS, univariate Cox regression was used to investigate SDGs with prognosis (Figure 3A and B). Then, using multivariate Cox regression, four genes (CXCL14, SLC44A1, UBD, and PPP2R2C) were found to be linked to survival (shown in Table 2). We identified that CXCL14 and SLC44A1 were significantly associated with DSS, and PPP2R2C and SLC44A1 were prognostic genes of PFS. Among these, CXCL14 and PPP2R2C were risk genes (HR>1). The SLC44A1 and UBD genes were protective in survival (HR<1). According to the prognostic gene expressions and their coefficient (17), we calculated the risk score [$\sum_{n=1}^{j}Coefj\;\;*\;\;Xj$, with Coefj indicating the coefficient and Xj representing the relative expression levels of each gene standardized by z-score] of each patient and used the median risk score value as a cut-off point for classifying the 30 high-grade HNC patients into a high-risk group and a low-risk group (n=15, respectively). DSS and PFS times in the high-risk group were shorter than that in the low-risk group (DSS, median time=1.431 years *vs* 2.625 years, P=0.033, Figure 4A; PFS, median time=1.361 years *vs* 2.261 years, P=0.010, Figure 4B).

**Table 2:** Significantly differential genes (SDGs) associated with prognosis in multivariate cox regression.

Gene	Coefficient	HR	95%CI	P value
CXCL14	5.728	307.226	1.271-74249.879	0.041*
SLC44A1	-21.232	0.000	0.000-0.612	0.045*
UBD	-2.036	0.131	0.009-1.852	0.132*
PPP2R2C	4.559	95.501	2.420-3769.563	0.015**
SLC44A1	-25.517	0.000	0.000-0.004	0.013**

Four SDGs were related with overall survival and used to calculate the risk score to classify the tumor patients into high- and low-risk groups. *DSS-related prognostic SDGs; **PFS-related prognostic SDGs. Risk score $\sum_{n=1}^{j}Coefj\;\;*\;\;Xj$; DSS: disease specific survival; PFS: progression free survival; HR: hazard ratio; CI: confidence interval.

### Prognostic hazard curves

**Figure 5:**
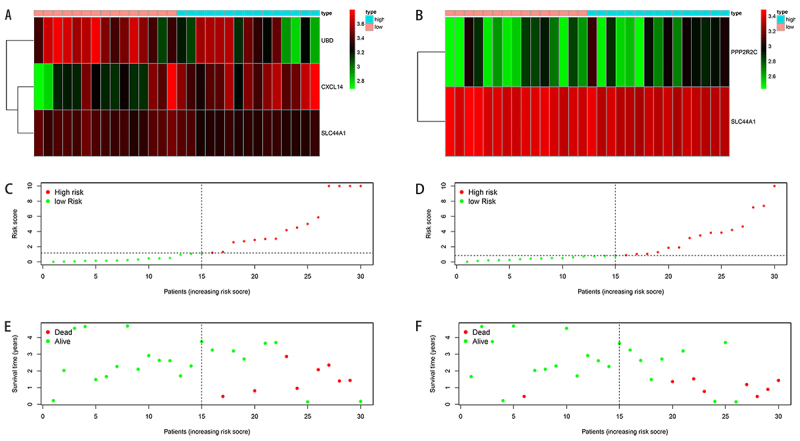
Risk score analysis based on the gene signature in the head and neck carcinomas (HNC) group. **A**, **C**, and **E**: Disease-specific survival (DSS); **B**, **D**, and **F**: progression-free survival (PFS). **A** and **B**: Heatmap of UBD, CXCL14, SLC44A1, and PPP2R2C expression in HNC samples. The colors from green to red indicate the expression level from low to high. **C** and **D**: Patient survival status and time distributed by risk score. The dotted line indicates the individual inflection point of the risk score curve, by which the patients were categorized into low-risk and high-risk groups. **E** and **F**: Risk score curve of the autophagy signature. The green dots represent patients who are alive and the red dots represent patients who have died.

We ranked the risk scores of patients for DSS and PFS and analyzed their survival distributions. For DSS, as the heatmap of risk score showed, patients with high-risk scores showed upregulation of CXCL14 and downregulation of UBD (Figure 5A). For PFS, patients with high-risk scores showed downregulation of PPP2R2C, implying it is a protective gene (Figure 5B). The dot plots showed the survival status of DSS and PFS of HNC patients (Figure 5C-F). When the risk score increased, the patients' risk increased and the survival time decreased.

### Independent risk factors of survival and ROC model

**Figure 6:**
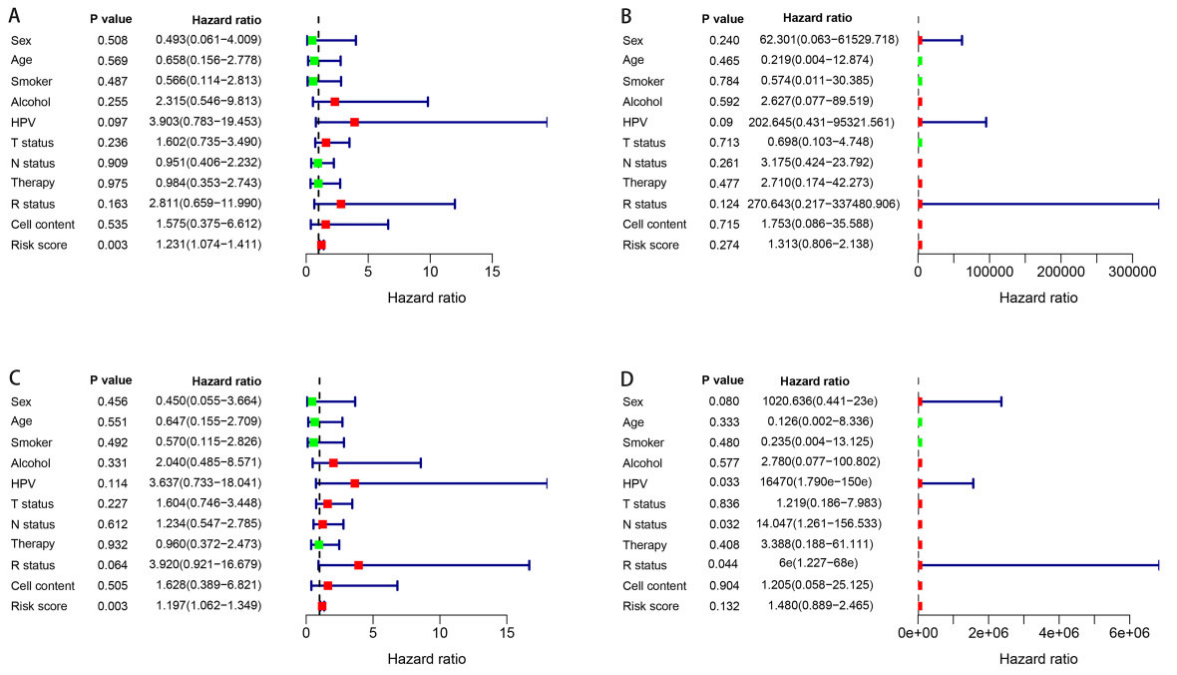
Univariate Cox regression forest plots of (**A**) disease-specific survival (DSS) and (**C**) progression-free survival (PFS). Multivariate Cox regression forest plots of (**B**) DSS and (**D**) PFS.

We combined the SDGs with clinical information in HNC patients. Univariate and multivariate Cox regression analyses were performed to investigate the independent risk factors for DSS and PFS. As shown in Figure 6A, univariate Cox regression showed the risk score was significantly associated with DSS (P=0.003). The multivariate regression showed there were no independent risk factors for DSS (all P>0.05) (Figure 6B). For PFS, the risk score was a significant risk factor in the univariate Cox regression (P=0.003) (Figure 6C). Multivariate Cox regression showed that HPV (P=0.033), lymph node status (P=0.032), and residual status (P<0.044) were independent risk factors for survival (Figure 6D).

**Figure 7:**
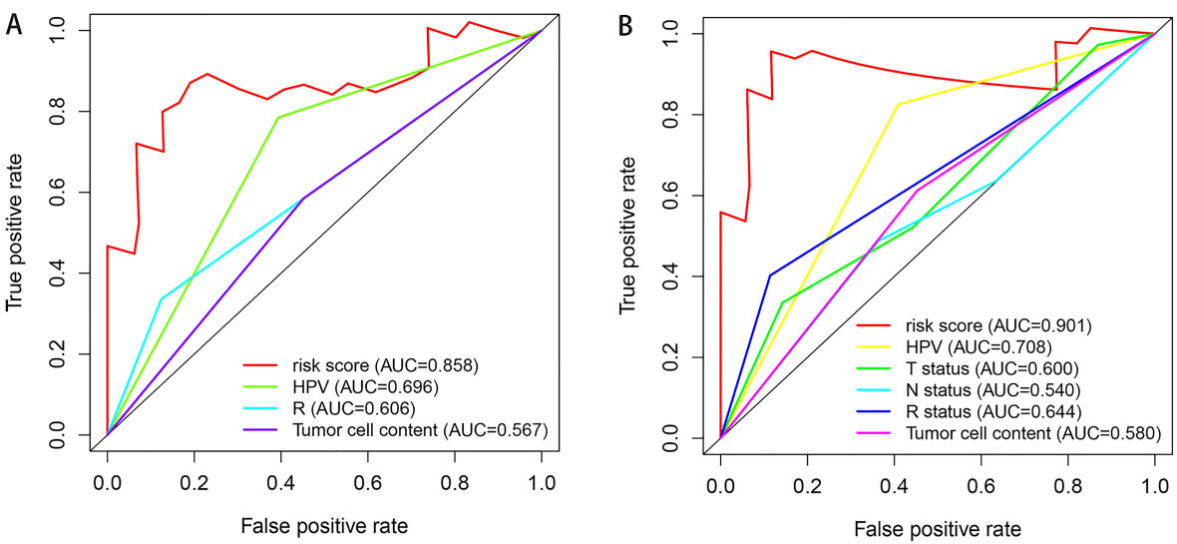
Prognostic performance of the risk factors models. ROC curves demonstrated the predictive abilities for (**A**) disease-specific survival (DSS) and (**B**) progression-free survival (PFS). The area under the curve (AUC) ranged from 0.5 to 1.0.

To provide a model to predict survival, we constructed the ROC curves using the risk factors associated with DSS and PFS. In addition, we assessed the feasibility using the area under the curve (AUC) values. Risk score, HPV, R, and tumor cell content were selected to establish the ROC, and the results showed the risk score had better ability to predict DSS (AUC=0.858) (Figure 7A). In the PFS analysis, five prognostic parameters, including the risk score, HPV, T, N, R, and tumor cell content, were recruited. The risk score performance showed better predictive ability than the other factors (AUC=0.901) (Figure 7B).

### Clinical correlation analysis

**Figure 8:**
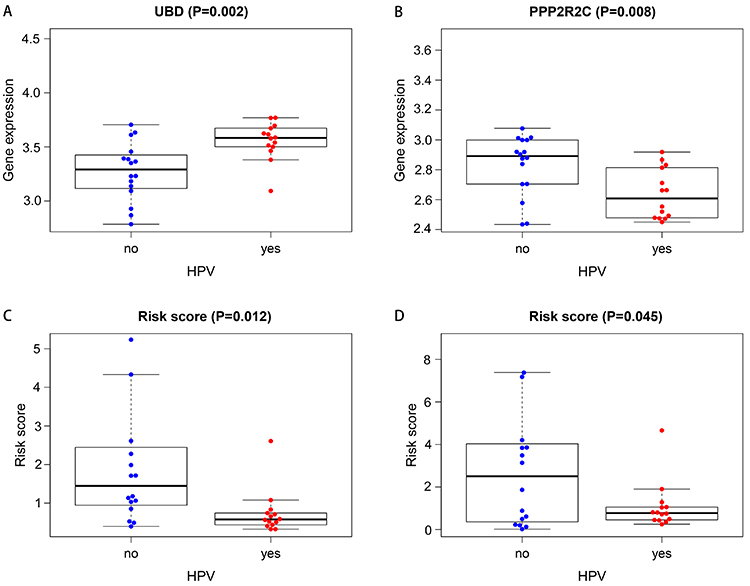
Correlations between significantly differential genes (SDGs) and clinical features. **A**, UBD expression level and human papillomavirus (HPV) status. **B**, PPP2R2C expression level and HPV status. Risk score level and the HPV status in the disease-specific survival (DSS) (**C**) and progression-free survival (PFS) (**D**) groups. Data are reported as medians and interquartile range (Student's *t*-test).

We further explored the relationships between the prognostic SDGs and clinical features. We calculated the correlations using the *t*-test or Kruskal-Wallis test. We found that UBD, PPP2R2C, and risk score were significantly associated with HPV status (all P values <0.05). UBD expression was higher in patients with HPV, and PPP2R2C expression was higher in patients with no HPV (Figure 8A and B). We also found risk score was significantly associated HPV status (Figure 8C and D).

### Experimental validation

**Figure 9:**
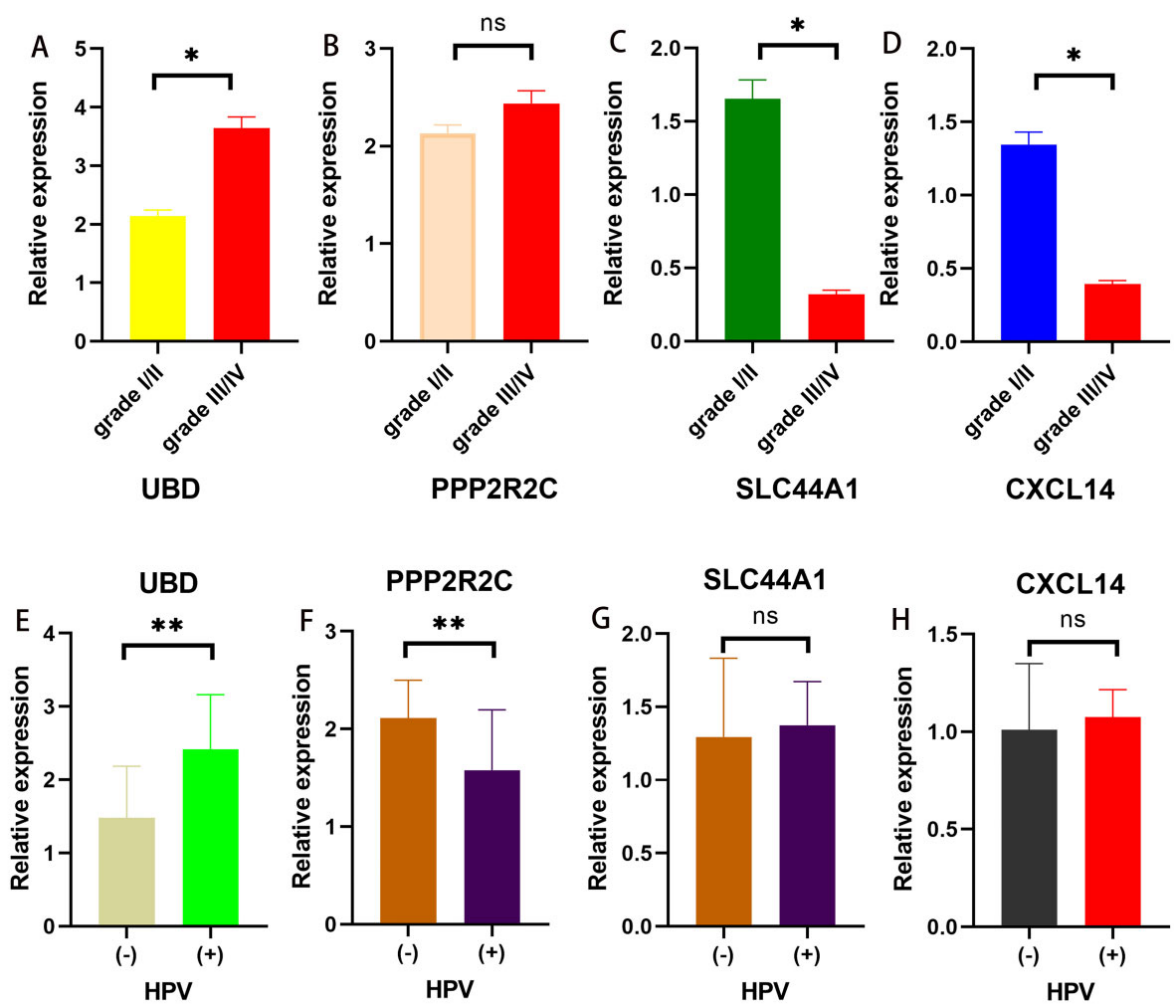
Four prognostic genes expression profiles and their correlations with human papillomavirus (HPV) status in the clinical tissue specimens. **A**, UBD; **B**, PPP2R2C; **C**, SLC44A1; and **D**, CXCL14 expression levels in the high-grade (III/IV) and low-grade (I/II) head and neck cancer (HNC). **E**, UBD; (**F**) PPP2R2C; (**G**) SLC44A1; and (**H**) CXCL14 expression levels in the HPV (+) and HPV (-) HNC patients. Data are reported as means±SD. *P<0.05, **P<0.01 (*t*-test). ns: not significant.

According to the screening and validation steps as described above, we performed experimental validation using the four prognostic genes (CXCL14, PPP2R2C, SLC44A1, UBD), and GAPDH was set as an internal reference. The results showed that CXCL14 and SLC44A1 were significantly overexpressed in HNC grade I/II tissues and UBD was overexpressed in HNC grade III/IV tissues. There was no significant difference in the expression levels of PPP2R2C between the two groups. The results are shown in Figure 9A-D.

In addition, we divided the 45 HNC patients into 21 HPV (+) and 24 HPV (-) groups according to their clinical HPV test results. Then, we further explored and verified the relationship between the four prognostic genes (CXCL14, PPP2R2C, SLC44A1, UBD) and HPV status. The results were in agreement to our bioinformatics analysis that UBD was significantly higher in the HPV (+) group and PPP2R2C was significantly higher in the HPV (-) group. The results are shown in Figure 9E and F.

## Discussion

Cancers are primarily caused by genetic alterations that result in the dysregulation of gene networks, which are responsible for malignancy. Numerous studies have now used high-throughput sequencing technology to profile different cancer samples. Current molecular studies of head and neck carcinomas focus primarily on the biological differences between the HPV-negative and -positive populations. Large consortiums have demonstrated that genes with frequent and significant genetic alterations are involved in various HNC cell functions, including tumor development and progression (18,19). However, few provide definitive evidence for elucidating the gene distinctions between low-grade and high-grade HNC. In this study, we found that low-grade and high-grade HNC have different gene expression profiles, which is directly linked to the DNA repair that may drive malignancy transformation from low- to high-grade. We also investigated the gene associations with clinical implications and discovered that SDGs were significantly related to DSS and PFS. To increase the reliability of the research, we confirmed these findings using our clinical tissue specimens.

We discovered that the SDGs were mainly enriched in the NF-kappaB signaling pathway and DNA repair by GO and KEGG analyses. Several studies have strongly supported the associations between the NF-kappaB signaling pathway and HNC (20-22). Qin et al. (20) found that CCL18 (chemokine (C‐C motif) ligand 18) could promote HNSCC, and its level was significantly associated with histological grade by regulating the NF-κB signal pathway. Yu et al. (21) provided evidence that the NF-κB pathway can be activated by CD147, which was positively correlated with HNSCC grade. Furthermore, the NF-κB inhibitor could reduce the invasion of HNSCC cells. In addition, the XPR1-induced NF-κB pathway was related to many aspects of tongue squamous cell carcinoma, including the tumor grade and patient prognosis (22). These studies have illuminated the functions of NF-κB signal pathway in HNSCC in terms of histological grade.

Continuous and chronic exposure to tobacco, alcohol, and infection with HPV are the predominant risk factors for HNC, which induce DNA damage (23). DNA repair mechanisms, such as excision repair, mismatch repair non-homologous end-joining, and homologous recombination protect genome against damage and provide stability for genes and chromosomes (23). Any low DNA repair efficacy is recognized as a mechanism for HNC initiation and progression. In addition, gene mutations and polymorphisms associated with DNA repair that HNC cells undergo are also determining factors promoting HNC (23). Much of the evidence of this comes from whole-exome sequencing studies. For example, exonic and intronic variants of several genes work together during the process of DNA repair, especially in the double-strand break repair and Fanconi anemia pathways (24). Moreover, it has been reported that certain genes involving the DNA repair pathways are correlated with HNC tumor size and clinical stage (25).

Instead of distinguishing genes directly through their associations with survival, we screened genes from different grades and then identified the prognostic genes. SLC44A1, also known as choline transporter-like 1 (CTL1), encodes an intermediate-affinity choline transporter protein. Choline is essential for all cells to synthetize the membrane phospholipids phosphatidylcholine (PC) and sphingomyelin and its uptake through SLC44A1 is strongly associated with cell viability, apoptosis, and malignant progression (26,27). SLC44A1 may be involved in the tumorigenesis and the metastasis of colon cancer, and is currently used as a prognostic biomarker (26). However, the field is still in its early stages and only a handful of studies have been conducted to assess the roles of SLC44A1 in HNC. Our experimental results demonstrated that SLC44A1 is upregulated in the low-grade (I/II) HNC, implying it may play a protective role in HNC. Nishiyama et al. (28) found that functional inhibition of CTL1 (SLC44A1) by cationic drugs could significantly increase caspase-3/7 activity and promote tongue cancer cell death. Identification of the CTL1-mediated choline transport system could provide a potential new target for tongue cancer therapy.

Another gene included in the prognostic model is PPP2R2C. This gene has been confirmed to be linked to gliomas, lung cancer, and prostate cancer, and it is thought to be a potential tumor-suppressor gene (29-31). Nonetheless, the role of PPP2R2C in our results has been questioned, which showed a lower expression in the high-grade group, implying a tumor-suppressor role. However, survival analysis showed a contradictory risk role (HR>1) in the prognosis. The discrepancy may be explained by the small number of samples. The determination of the role of PPP2R2C in HNC necessitates experimental analysis that will delineate the contribution of PPP2R2C in the function of HNC cells. Our experimental result confirmed the slightly higher level of PPP2R2C in high-grade HNC, implying it may serve as a tumor-promoting gene, but without significant statistical difference (P>0.05). Previous research demonstrated that PPP2R2C is subjected to transcriptional regulation by factors such as miRNAs, which are involved in HNC cell proliferation, invasion, and recurrence (32,33). However, the mechanisms of miRNAs on cancer cell activities through regulating the expression of PPP2R2C need to be further investigated.

To verify whether the prognostic risk factors could predict survival, we further established a ROC model using the factors selected from multivariate Cox regression. The risk score showed excellent predictive ability, implying it could serve as an accurate survival indicator both for DSS and PFS (AUC=0.0858 and 0.901 respectively). To gain a better understanding of how genes influence the clinical characteristics, we assessed the relationships between the SDGs and clinical features in HNC patients. UBD, PPP2R2C, and risk score were strongly associated with HPV status (all P<0.05). Wang et al. (34) reported that the UBD expression level was much higher in the HPV (+) oropharyngeal squamous cell carcinoma (OSCC) compared with the HPV (-) OSCC. Our results are congruent with their findings. We also found that the PPP2R2C level was higher in the HPV (-) group than that in the HPV (+) group. However, no more studies have investigated the correlation between PPP2R2C and HPV until now. Further research is needed to explore the associations. In addition, these may be critical to understand the cause of differences in clinical presentation and molecular landscapes and develop tailored therapy for the HPV (+) and HPV (-) HNC.

The strength of our study is that we performed a systematic analysis to identify SDGs in different HNC grades using a public database, with experimental validation. This work may help shed light on HNC malignant progression and develop new targeted drugs. Several limitations should be addressed in this study. Firstly, the sample number was too small to reach a robust conclusion that applies to all tumors of the head and neck. Secondly, the exact mechanisms by which SDGs drive malignancy transformation from low-grade to high-grade are still unknown. Lastly, we failed to examine the significance of SDGs for all clinical implications, such as therapy modality. Notwithstanding its limitations, this study provided a preliminary overview of SDGs profile in HNC and the limitations can be solved if there are more functional validations and translations into clinical implications in the future.

In conclusion, we identified different SDGs expression profiles between the high-grade and low-grade HNC by analyzing a public database and conducting an experiment. This study indicated the prognostic genes and survival of HNC from the perspectives of bioinformatics. However, further validations are needed to confirm the findings of our study.

## Supplementary Material

Click here to view [pdf].
